# Plasma Exosome miRNAs Profile in Patients With ST-Segment Elevation Myocardial Infarction

**DOI:** 10.3389/fcvm.2022.848812

**Published:** 2022-06-15

**Authors:** Ruicong Guan, Kuan Zeng, Bin Zhang, Minnan Gao, Jianfen Li, Huiqi Jiang, Yuqiang Liu, Yongjia Qiang, Zhuxuan Liu, Jingwen Li, Yanqi Yang

**Affiliations:** ^1^Department of Cardiovascular Surgery, Sun Yat-sen Memorial Hospital, Sun Yat-sen University, Guangzhou, China; ^2^Guangdong Provincial Key Laboratory of Malignant Tumor Epigenetics and Gene Regulation, Sun Yat-sen Memorial Hospital, Sun Yat-sen University, Guangzhou, China; ^3^Department of Cardiothoracic Surgery, University Hospital, Linköping University, Linköping, Sweden

**Keywords:** exosome, miRNA, myocardium remodeling, STEMI, post infarction

## Abstract

**Background:**

Circulating microRNAs (miRNAs) have been found to have different expressions in different phases of acute myocardial infarction. The profiles of plasma exosome miRNAs in patients with ST-segment elevation myocardial infarction (STEMI) at 3–6 months postinfarction are unknown.

**Objective:**

The aim of this study was to assess the profiles of plasma exosome miRNAs in patients with STEMI in comparison with healthy volunteers and to select specific exosome miRNAs related to pathophysiological changes post-STEMI.

**Methods:**

Plasma and echocardiography parameters were collected from 30 patients 3–6 months after STEMI and 30 healthy volunteers. Plasma exosome miRNAs were assessed by using high-throughput sequence (Illumina HiSeq 2500) and profile of the plasma exosome miRNAs was established in 10 patients and 6 healthy volunteers. The specific exosome miRNAs related to heart diseases were selected according to the TargetScan database. The specificity of the selected exosome miRNAs was evaluated in additional 20 post-STEMI patients and 24 healthy volunteers by using quantitative PCR (qPCR). Left ventricular remodeling (LVR) was defined using the European Association of Cardiovascular Imaging criteria according to echocardiography examination. Correlations between expression of the specific miRNAs and echocardiography parameters of LVR were assessed using the Spearman correlation analysis.

**Results:**

Twenty eight upregulated miRNAs and 49 downregulated miRNAs were found in patients 3–6 months after STEMI (*p* < 0.01) in comparison with the healthy volunteers. The two least expressed and heart-related exosome miRNAs were hsa-miR-181a-3p (0.64-fold, *p* < 0.01) and hsa-miR-874-3p (0.50-fold, *p* < 0.01), which were further confirmed by using qPCR and demonstrated significant specificity in another 20 patients with post-STEMI comparing to 24 healthy volunteers [area under the curve (AUC) = 0.68, *p* < 0.05; AUC = 0.74, *p* < 0.05]. The expression of hsa-miR-181a-3p was downregulated in patients with LV adverse remodeling in comparison with patients without LV adverse remodeling and healthy volunteers.

**Conclusion:**

Circulating exosome miR-874-3p and miR-181a-3p were downregulated in patients with STEMI postinfarction. Exosome hsa-miR-181a-3p might play a potential role in the development of LVR in patients with post-STEMI.

## Introduction

ST-segment elevation myocardial infarction (STEMI) is a fatal cardiovascular disease (1). The incidence of STEMI in European countries is approximately 0.04–0.14% per year (2). STEMI is caused by ruptured epicardial atherosclerotic plaque with activation of thrombosis, leading to necrosis of myocytes and denaturation of collagen fibers (3, 4). Left ventricular remodeling occurs during the period of postinfarction, characterized by a rise in left ventricular end-diastolic volume (LVEDV) and a relative reduction of left ventricular ejection fraction (LVEF) (5). In fact, one-half of patients with STEMI at 3–6 months postinfarct were likely to develop symptomatic heart failure (6).

The wide use of pharmacotherapy and revascularization has been associated with a rise of survival rate in patients with STEMI at the early stage of STEMI (7, 8). Few studies focused on biochemical parameters to predict the prognosis of myocardial remodeling at the late stage (9), specifically in patients with STEMI 3–6 months after survival from the onset of the disease (10, 11). In light of these findings, there is a need to identify biophysical parameters that reflect the pathological changes of STEMI for better prognosis (12).

Circulating microRNAs (miRNAs) are one of the important non-coding RNAs encoded by endogenous genes with a length of 22 nucleotides (nts), taking part in posttranscriptional regulation of messenger RNA (mRNA) expression (13). Exosomal miRNAs are more stable biomarkers than non-exosomal miRNAs in controlling posttranscription cardiac gene expression in a paracrine manner to promote angiogenesis (14). Additionally, the regulatory role of exosome miRNAs in the pathogenicity, development, and therapeutic management of cardiac diseases has been widely accepted (15). However, it is unclear regarding which exosome miRNAs were dysregulated postinfarction and which were closely related to left ventricular remodeling.

Therefore, in this study, the expression levels of exosome miRNAs were quantified using high-throughput sequencing with validation of their sensitivity in patients with STEMI at 3–6 months postinfarct compared with healthy volunteers using quantitative PCR (qPCR) and determination of whether these exosome miRNAs were related to clinical data such as N-terminal pro-brain natriuretic peptide (NT-proBNP), lactate dehydrogenase (LDH), and Doppler echocardiography parameters such as LVEF and LVEDV.

## Materials and Methods

### Ethics Statement, Echocardiographic Measurements, and Plasma Collection

This study was performed in accordance with the Ethics Committee of Sun Yat-sen Memorial Hospital (SYSEC-KY-KS-2020-191). Volunteers of young ages were selected as healthy volunteers for ruling out the development of risk factors for STEMI and patients with STEMI 3–6 months postinfarct were recruited into the ischemic heart disease (IHD) group. Patients with STEMI events were older than the healthy controls, as previously reported (16). The training group comprised 16 subjects and the internal validation group comprised 44 subjects. By comparison between healthy volunteers and patients, no statistically significant differences were found in sex and other considered variables. The inclusion criteria included individuals aged from 40 to 80 years who have been diagnosed with STEMI according to ECG results and myocardial enzymes. The exclusion criteria included malignant tumors, renal transplantation, and myocarditis. Informed consent was obtained from all the study subjects. Patients with STEMI with ≥ 20% increase in LVEDV and ≥ 5% decrease in LVEF from baseline were considered to have adverse structural LV remodeling (17). Transthoracic echocardiography was assessed using 2-dimensional imaging (Philips EPIQ Elite) according to the recommendations for cardiac chamber quantification (18, 19). The view of left ventricle from either the patients or healthy volunteers was placed in the left supine position by the parasternal long axis.

From 26-10-2020 to 19-1-2021, 3–6 months after the onset of STEMI symptoms, plasma samples were obtained from whole-blood samples of the test subjects within 1 h for centrifugation. Plasma was collected into free-enzyme-containing ethylenediaminetetraacetic acid (EDTA) tubes for centrifugation at 3,000 *g* for 10 min and then the supernatant was centrifugated at 13,000 *g* and transferred to 13.2 ml Beckman tubes and centrifugated at 10,000 *g* for 10 min and 100,000 *g* for 70 min at 4°C. Finally, the precipitate was collected into 1.5 ml Eppendorf (EP) tubes and stored at −80°C for further analysis. Among total plasma samples, for the training group, plasma samples from 10 patients and 6 healthy volunteers were analyzed by high-throughput sequencing (Illumina HiSeq 2000/2500) and for the validation group, plasma samples from another 20 patients and 24 healthy volunteers were tested by qPCR.

### Exosome Isolation and Detection

Exosomes were purified from the plasma using ultracentrifugation (Hitachi, #CP100MX) according to the recommended protocol. The morphology of the exosome was visualized with transmission electron microscopy (Hitachi, #HT-7700) at 100 kV. Nanoparticles were detected by high-sensitivity flow cytometry (NanoFCM N30E). Exosome (10 μl) was diluted to 30 μl for aspiration, with attention focused on the need for gradient dilution to avoid sample clogging of the injection needle.

#### Western Blotting

The expressions of TSG101, CD81, and glyceraldehyde-3-phosphate dehydrogenase (GAPDH) in exosome and exosome-free protein were measured using a standard Western blot assay. Equivalent amounts of proteins (10 μg/μl) measured by the Bicinchoninic Acid (BCA) Protein Assay Kit were boiled with sodium dodecyl sulfate-polyacrylamide gel electrophoresis (SDS-PAGE) loading buffer at 95°C and then subjected to 10% SDS-PAGE before being transferred onto cropped 0.22 μm polyvinylidene difluoride (PVDF) membranes (Servicebio, Wuhan, China) at 200 mA for 1.5 h. After that, the membranes were incubated at 4°C for 24 h with primary antibodies against anti-TSG101 (1:500, Servicebio, #GB11619), CD81 (1:500, Servicebio, #GB111073), and GAPDH (1:1,000, Servicebio, #GB11002). Next, the membranes were incubated with horseradish peroxidase (HRP)-conjugated secondary goat antirabbit (1:2,000, Servicebio, #GB23303) at room temperature for 1 h. Enhanced Chemiluminescence (ECL) Reagent Kit was used for the detection of proteins on the blotting membranes.

### Exosome microRNA High-Throughput Sequence Analysis

Exosome miRNA sequencing was performed on 16 plasma samples (10 patients with STEMI and 6 healthy volunteers) for the test group. The small RNA sequencing library was prepared using the TruSeq Small RNA Sample Prep Kits (Illumina, San Diego, California, United States). RNA integrity was then examined through the BioAnalyzer 2100 (Agilent, California, United States). Approximately, 100 ng of RNA were used to prepare a small RNA library according to the protocol of the TruSeq Small RNA Sample Prep Kits (Illumina, San Diego, California, United States). Then, the single-end sequencing (1 bp × 50 bp) was performed on Illumina HiSeq 2500 by LC-Bio (Hangzhou, China) following the vendor’s recommended protocol. The raw data of high-throughput sequencing was deposited at the GSE 185729 in the National Center for Biotechnology Information (NCBI).

### Identification of microRNA

Raw data was processed by an in-house program, ACGT101-miR (LC Sciences, Houston, Texas, United States), to remove adapter dimers, junk, low complexities, common RNA families [ribosomal RNA (rRNA), transfer RNA (tRNA), small nuclear RNA (snRNA), and small nucleolar RNA (snoRNA)], repeats, and sequences < 18 or > 26 nt in length. Mapping was also performed on pre-miRNA against human genomic data. Unique sequences with a length of 18 to 26 nt were mapped to miRNA sequences in miRBase 22.0. The unique sequences that aligned to the known miRNA sequences in miRBase 22.0 were identified as known miRNA. To identify the results of putative miRNAs in humans, all the obtained miRNAs were used to predict the secondary structures using RNA fold software.

### Analysis of Differentially Expressed microRNAs

The differential expression of miRNAs based on normalized deep-sequencing counts was analyzed by selectively using the Fisher’s exact test, Student’s *t*-test, or ANOVA. The significance α threshold was set to be 0.05 in all the statistical tests.

### Prediction of Target Genes of microRNAs

To predict the genes targeted by differentially expressed miRNAs, TargetScan 5.0, miRanda 3.3, and miRPath v3.0 were used to identify miRNA-binding sites. Finally, the data predicted by both the algorithms were combined and the overlaps were calculated. The Gene Ontology (GO) terms and the Kyoto Encyclopedia of Genes and Genomes (KEGG) pathway of these differentially expressed miRNA targets were also annotated.

### Exosome RNA Extraction for Quantitative PCR

**FIGURE 1:**
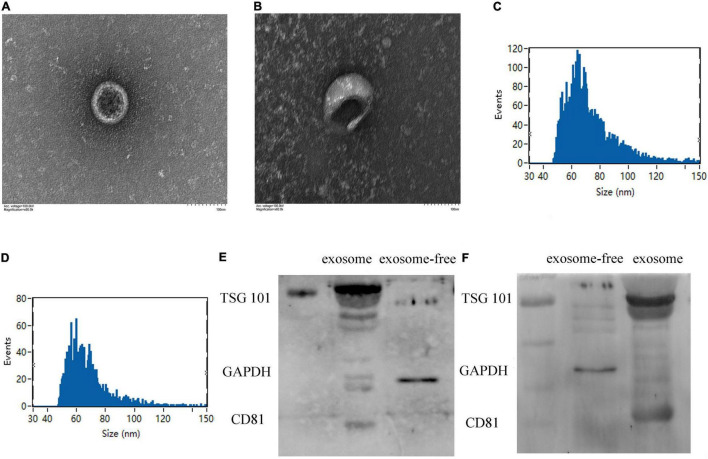
Identification of plasma exosome using transmission electron microscopy (TEM) and Western blotting. **(A,B)** The specific morphology of plasma exosome in patients **(A)** and in healthy volunteers **(B)** was identified by TEM. **(C,D)** NanoFCM showed that the diameter of exosome ranged from 50 to 70 nm in patients **(C)** and in healthy volunteers **(D)**. **(E,F)** Compared to exosome-free protein, Western blot analysis shows that exosomes were positive for TSG101 and CD81 and negative for glyceraldehyde-3-phosphate dehydrogenase (GAPDH) in healthy volunteers **(E)** and in patients **(F)**.

Total RNA was extracted from blood plasma samples using the Exosomal RNA Isolation Kit (Norgen, #5800) in line with the manufacturer’s protocol. Analyzed data of the total RNA quantity and purity are shown in Figure 1. Expression levels of hsa-miR-181a-3p (ACCATCGACCGTTGATTGTACC) and hsa-miR-874-3p (CTGCCCTGGCCCGAGGGACCGA) were determined. We then correlated plasma exosome miRNAs with LDH, NT-proBNP, LVEF, LVEDV, and troponin I (TnI) measured at 3–6 months postinfarction using the Spearman correlation R method.

### Statistical Analysis

The data were analyzed using the SPSS version 17.0 (SPSS Incorporation, Chicago, Illinois, United States) or the GraphPad Prism version 7 software (GraphPad Software Incorporation, La Jolla, California, United States) and were presented as mean ± SD. Measurement data with normal distribution were compared between the 2 groups using the Student’s *t*-test and *p* < 0.05 was considered as statistically significant.

## Results

### Clinical Characteristics of Patients With ST-Segment Elevation Myocardial Infarction at 3–6 Months Postinfarct

MicroRNAs were extracted from plasma exosomes of 30 patients with STEMI and 30 healthy volunteers. The clinical characteristics of patients with STEMI after 3–6 months are given in Table 1 and Supplementary Table 1.

**TABLE 1:** Characteristics of patients 3–6 months after ST-segment elevation myocardial infarction (STEMI) and healthy volunteers (health).

Study variables	Health (*n* = 30)	STEMI (*n* = 30)	*p*
Mean age, years (SD)	32.37, (0.86)	64.71, (10.40)**	0.01
Males, *N* (%)	25, 83 (%)	27, 90 (%)	0.45
Weight (kg)	70.78, (1.70)	65.64, (1.81)	0.24
Height (cm)	165, (4.06)	166, (1.13)	0.83
ALT	18.67, (4.31)	30.00, (7.03)	0.26
AST	22.33, (2.90)	38.67, (12.75)	0.20
Cr	94, (5.53)	131.5, (19.43)	0.06
Triglyceride	1.59, (0.35)	1.43, (0.14)	0.67
Total cholesterol	4.41, (0.21)	4.50, (0.36)	0.83
HDL-C	1.18, (0.04)	0.93, (0.04) **	0.00
LDL-C	2.83, (0.14)	3.01, (0.29)	0.58
NT-proBNP	< 10	5985	−
LDH	160.9, (4.15)	250.1, (18.67)**	0.00
LVEF (%)	66.93, (0.67)	41.5, (15.36) **	0.00
LVEDV (ml)	111.6, (2.49)	149.4, (11.24) **	0.00

ALT, Alanine aminotransferase; AST, Aspartate aminotransferase; Cr, Creatinine; HDL-C, High-density lipoprotein cholesterol; LDL-C, Low-density lipoprotein cholesterol; LVEF, Left ventricular ejection fraction; LVEDV, LV end-diastolic volume.Comparison between STEMI patients and healthy volunteers. **p < 0.01 versus healthy volunteers.[Data are presented as mean, (Std. Deviation)].

### Identification of Exosomes Extracted From Plasma

Exosomes extracted from plasma were identified by transmission electron microscopy (Figures 1A,B), displaying typical shapes, including centrally concave, disk-shaped bilayers with a size of 50–70 nm with a concentration of 1.24E + 10 to 7.66E + 10 calculated by NanoFCM N30E (Figures 1C,D). Exosomal protein biomarkers were measured by Western blot assay. Compared to exosome-free protein, TSG101 and CD81 were positive and GAPDH was negative in protein lysis isolated from human plasma exosome (Figures 1E,F).

### Exosome microRNA Expression Profiling and Bioinformatics Analysis From Patients 3–6 Months After ST-Segment Elevation Myocardial Infarction and Healthy Volunteers

**FIGURE 2:**
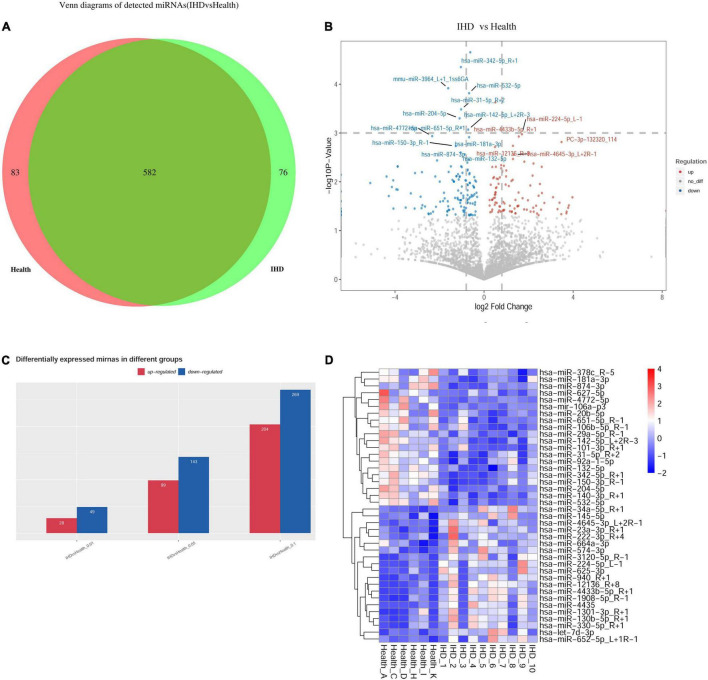
Profile of exosomal microRNAs (miRNAs) using high-throughput sequencing. **(A)** Venn diagram showed overlapping miRNAs between healthy volunteers and patients, where 76 miRNAs were specifically detected in ST-segment elevation myocardial infarction (STEMI). While 83 miRNAs were specifically detected in healthy volunteers, 582 miRNAs showed no statistically significant difference between them. **(B)** The overall distribution of differentially expressed miRNAs was shown in volcano plots using log2(fold change) as the horizontal coordinate and –log10 (*p*-value) as the vertical coordinate. **(C)** Bar plot: Differentially expressed miRNA was observed, including 28 upregulated miRNAs and 49 downregulated miRNAs (*p* < 0.01). **(D)** Heatmap in patients with STEMI after 3–6 months (*n* = 10) and healthy volunteers (*n* = 6) uncovered the similarity of miRNA expression profiles of the discovery group by clustering miRNAs, where the top 40 dysregulated miRNAs (*p* < 0.05) are shown on the right.

High-throughput sequencing (Illumina HiSeq 2000/2500) was utilized to detect the plasma exosome miRNAs from exosome expression profiles in the training group of 10 patients with STEMI 3–6 months postinfarction and 6 healthy volunteers. Venn diagrams revealed that 76 miRNAs were specifically detected in patients with STEMI 3–6 months postinfarction, while 83 miRNAs were specifically detected in healthy volunteers, in addition to 582 miRNAs being overlapped between them (Figure 2A). The overall distribution of differentially expressed miRNAs was shown in volcano plots using log2(fold change) as the horizontal coordinate and -log10 (*P*-value) as the vertical coordinate (Figure 2B); the bar plot comprised differentially expressed miRNA (*p* < 0.01), including 28 upregulated miRNAs and 49 downregulated miRNAs (Figure 2C); hierarchical clustering heat map uncovered the similarity of miRNA expression profiles in the discovery group by clustering the top 40 dysregulated miRNAs (Figure 2D).

### Annotations of the Selected microRNA Target Genes and Enrichment of Their Signaling Pathway

**FIGURE 3:**
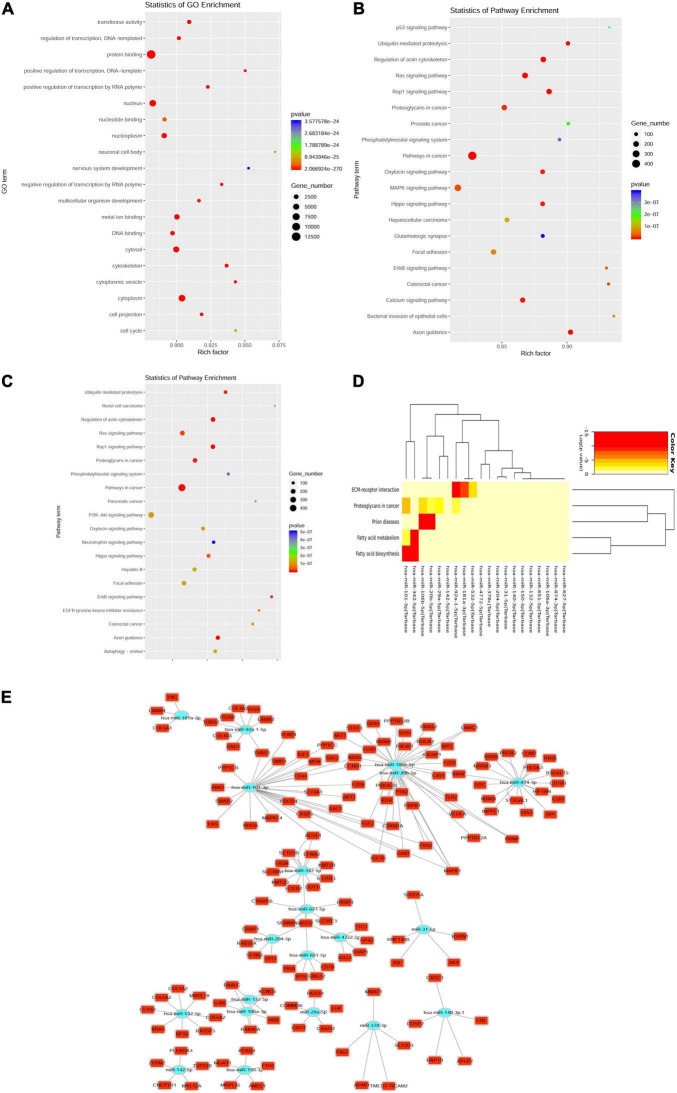
Top 40 dysregulated miRNAs targeting messenger RNA (mRNA). **(A)** Enrichment of the Gene Ontology (GO) annotated mRNAs (dysregulated miRNAs targeting mRNA). **(B,C)** Enrichment of upregulated miRNAs targeting mRNAs in the Kyoto Encyclopedia of Genes and Genomes (KEGG) pathway **(B)**; Enrichment of downregulated miRNAs targeting mRNAs in the KEGG pathway **(C)**. Y-axis label represents the pathway and X-axis label represents the rich factor. The greater the rich factor, the greater the degree of pathway enrichment. The size and color of the bubble represents the amount of differentially expressed genes enriched in the pathway and enrichment significance, respectively. **(D)** Heatmap of downregulated miRNA targeting the KEGG pathway clusters using miRPath v3 bioinformatics tools (top 20 downregulated miRNAs targeting mRNA). **(E)** The network indicating the top 20 downregulated miRNAs targeted genes.

Among all the selected miRNA target genes, the Gene Ontology (GO) annotations were classified in correspondence to these genes into three categories (molecular function, biological process, and cellular component) and the GO functions in each category were sorted according to the number of annotated target genes. The percentage of the number of target genes annotated to the same GO can be visualized (Figure 3A). Upregulation of exosome miRNA targeting genes, which were closely associated with heart diseases, was enriched in the p53 signaling pathway, ubiquitin-mediated proteolysis, regulation of actin cytoskeleton, renin-angiotensin system (RAS) signaling pathway, Rap1 signaling pathway, phosphatidylinositol signaling system, mitogen-activated protein kinase (MAPK) signaling pathway, glutamatergic synapse, and calcium signaling pathway (Figure 3B); downregulation of exosome miRNA targeting genes was enriched in ubiquitin-mediated proteolysis, regulation of actin cytoskeleton, RAS signaling pathway, Rap1 signaling pathway, phosphatidylinositol signaling system, phosphatidylinositol-3-kinase (PI3K)-Akt signaling pathway, neurotrophin signaling pathway, focal adhesion, ErbB signaling pathway, epidermal growth factor receptor (EGFR) tyrosine kinase inhibitor resistance, axon guidance, and autophagy (Figure 3C). Statistics of pathway enrichment indicated that the rich factor of the p53, Ras, and Rap1 signaling pathways was over 0.9 in the upregulation of exosome miRNAs. Interestingly, the Ras signaling pathway, Rap1 signaling pathway, and phosphatidylinositol signaling system were enriched in both the dysregulated exosome miRNAs targeting genes in the KEGG signaling pathway, which ascertained that they were critical regulators in the pathogenesis of myocardial infarction. Blocking the Ras signaling pathway could at least retard the progression of heart failure using mono or dual therapy (20).

Among the top 40 dysregulated exosomal miRNAs, the top 20 targeted downregulated miRNAs were enriched in fatty acid biosynthesis and metabolism (Figure 3D). This was performed using miRPath v3.0 with previous experimental support (21). Aerobic fatty acid biosynthesis and metabolism were impaired, while the glycolytic system was augmented during myocardial ischemia (22). As shown in the network plot (Figure 3E), miR-181a-3p targeting genes were tenascin C (TNC), laminin subunit alpha 4 (LAMA4), collagen type V alpha 1 chain (COL5A1), and thrombospondin 2 (THBS2). MiR-874-3p targeting genes were dopa decarboxylase (DDC), catechol-*O*-methyltransferase (COMT), estrogen receptor 1 (ESR1), thyroid hormone receptor alpha (THRA), retinoid X receptor beta (RXRB), ST3 beta-galactoside alpha-2,3-sialyltransferase 1 (ST3GAL1), beta-1,3-galactosyltransferase 5 (B3GALT5), B-Raf proto-oncogene, serine/threonine kinase (BRAF), inositol polyphosphate phosphatase-like 1 (INPPL1), inhibitor of nuclear factor kappa-B kinase subunit beta (IKBKB), CD79b molecule (CD79B), and vav guanine nucleotide exchange factor 3 (VAV3).

### Validation of the Exosome microRNAs Through Quantitative PCR Test and Spearman Correlation Analysis

We included 20 patients with STEMI 3–6 months postinfarction and 24 healthy volunteers as the validation group to verify RNA sequencing (RNA-seq) data. Results included 25 upregulated miRNAs and 13 downregulated miRNAs, which were possible with confirmed relation to cardiovascular diseases (Table 2). U6 was chosen as the miRNA control to normalize the expression of exosome miRNA.

**TABLE 2:** List of exosomal microRNA (miRNA) candidates (*p* < 0.05) from high-throughput sequencing.

miRNA	Up/down	Fold change (IHD *n* = 10/Health *n* = 6)
hsa-miR-140-3p	down	0.64
hsa-miR-532-5p	down	0.61
hsa-miR-31-5p	down	0.52
hsa-miR-204-5p	down	0.47
hsa-miR-181a-3p	down	0.64
hsa-miR-874-3p	down	0.50
hsa-miR-132-5p	down	0.47
hsa-miR-20b-5p	down	0.56
hsa-miR-29a-5p	down	0.42
hsa-mir-106a-p3	down	0.44
hsa-miR-106b-5p	down	0.75
hsa-miR-101-3p	down	0.53
hsa-miR-193a-3p	down	0.49
hsa-miR-4443	down	0.14
hsa-miR-362-5p	down	0.71
hsa-miR-320	down	0.55
hsa-miR-363-3p	down	0.57
PC-3p-56879_271	down	0.26
hsa-miR-542-3p	down	0.57
hsa-miR-107	down	0.72
hsa-miR-6815-5p	down	0.36
hsa-miR-873-5p	down	0.28
hsa-miR-542-3p	down	0.57
hsa-miR-107	down	0.72
hsa-miR-6815-5p	down	0.36
hsa-miR-873-5p	down	0.28
hsa-miR-23a-3p	up	1.33
hsa-miR-224-5p	up	3.15
hsa-let-7d-3p	up	1.34
hsa-miR-940	up	4.48
hsa-miR-3120-5p	up	4.03
hsa-miR-664a-3p	up	1.21
hsa-miR-34a-5p	up	2.18
hsa-miR-652-5p	up	1.71
hsa-miR-625-3p	up	2.03
hsa-miR-145-5p	up	1.67
hsa-miR-505-3p	up	1.36
hsa-miR-128-3p	up	1.36
hsa-miR-505-5p	up	1.38

**FIGURE 4:**
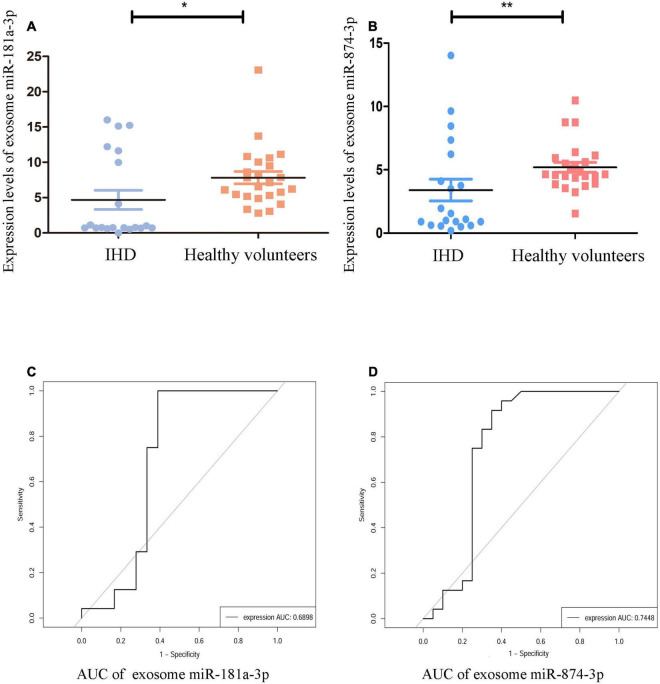
Validation of specific exosomal miRNAs. The validation group comprised 20 patients with STEMI and 24 healthy volunteers. The relative expression levels of exosome miRNAs were normalized to levels of U6. **(A)** Compared to healthy volunteers (*n* = 24), expression of hsa-miR-181a-3p was significantly reduced in patients with STEMI [ischemic heart disease (IHD), *n* = 20] and was normalized to levels of U6 using quantitative PCR (qPCR), **p < 0.01. **(B)** Compared to healthy volunteers (*n* = 24), expression of hsa-miR-874-3p was significantly lowered in patients with STEMI (IHD, *n* = 20) and was normalized to levels of U6 using qPCR, **p < 0.01. **(C)** The receiver operating characteristic (ROC) curve of hsa-miR-181a-3p. **(D)** The ROC curve of hsa-miR-874-3p. *p < 0.05.

**FIGURE 5:**
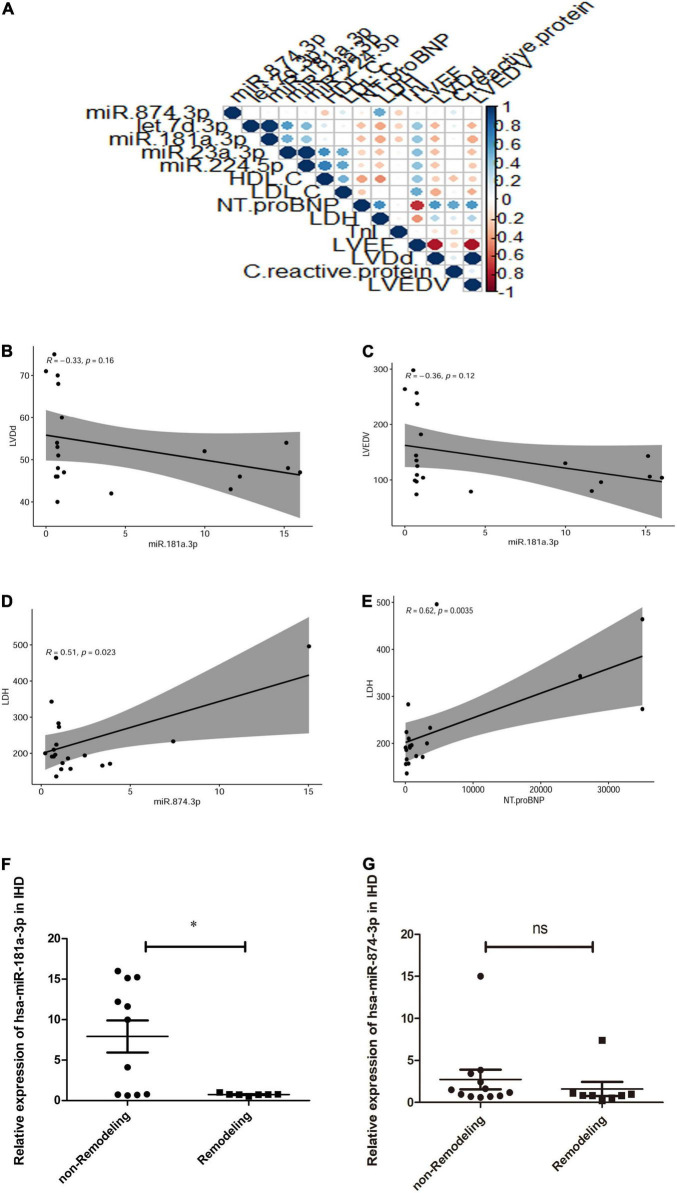
Hierarchical clustering heatmap and the correlation curves of exosome miRNA expression across clinical biomarker levels in patients. **(A)** Hierarchical clustering heatmap of expression of five exosome miRNAs across clinical biomarker levels in patients (*n* = 20) and the correlation of biomarker levels is presented with red (positive) or blue color (negative). Y-axis represents the Rho correlation coefficient and X-axis represents clinical data, such as high-density lipoprotein-cholesterol (HDL-C), low-density lipoprotein-cholesterol (LDL-C), N-terminal pro-brain natriuretic peptide (NT-proBNP), lactate dehydrogenase (LDH), troponin I (TnI), left ventricular ejection fraction (LVEF), left ventricular diastolic diameter (LVDd), left ventricular end-diastolic volume (LVEDV), and expression of circulating exosome miRNAs, including miR-874-3p, Let-7d-p, miR-181a-3p, miR-23a-3p, and miR-224-5p. **(B)** Correlation between miR-181a-3p expression and LVEDV (*p* = 0.16, *r* = –0.33). **(C)** Correlation between miR-181a-3p expression and LVDd (*p* = 0.12, *r* = –0.36). **(D)** Plasma exosome miR-874-3p exhibited a positive correlation with LDH (*p* < 0.05, *r* = 0.50). **(E)** Levels of LDH were positively associated with NT-proBNP (*p* < 0.01, *r* = 0.62) in patients with STEMI 3–6 months postinfarction. **(F)** Expression of miR-181a-3p was downregulated in patients with adverse LV remodeling in comparison with patients without LV adverse remodeling. **(G)** Relative expression of miR-874-3p in patients with and without adverse LV remodeling. *p < 0.05, ns means *p*> 0.05.

As shown in Figures 4A,B, in comparison with healthy volunteers, the relative expression of exosome miR-874-3p and miR-181a-3p showing downregulation in STEMI after 3–6 months was consistent with high-throughput sequencing. The area under the curve (AUC) values of miR-181a-3p and miR-874-3p were 0.68 and 0.74, respectively (Figures 4C,D). Figure 5A illustrates the hierarchical clustering heat map of five exosome miRNA expression across clinical data levels in patients. As shown in Figures 5B,C, the correlation between expression of miR-181a-3p and LVEDV or left ventricular diastolic diameter (LVDd) showed no statistical significance. As shown in Figure 5D, exosome miR-874-3p exhibited a positive correlation with LDH (*p* < 0.05, *r* = 0.50). It was found that the serum levels of LDH served as a predictor of adverse LV remodeling in a former study (23). As shown in Figure 5E, levels of LDH were positively associated with NT-proBNP (*p* < 0.01, *r* = 0.62) in patients with STEMI after 3–6 months. As given in Figure 5F, the expression of miR-181a-3p was downregulated in patients with adverse LV remodeling and there was a significant difference. As shown in Figure 5G, no significant differences in the expression of miR-874-3p were found in patients with and without LV adverse remodeling. However, there were no significant differences in the expressions of hsa-miR-181a-3p between patients without LV adverse remodeling and healthy individual (*p* = 0.95), as shown in Supplementary Figure 1A. Furthermore, as shown in Supplementary Figure 1B, expression levels of hsa-miR-181a-3p showed significant difference between patients with LV adverse remodeling and patients without LV adverse remodeling (AUC = 0.79, *p* = 0.03). However, expression levels of hsa-miR-874-3p cannot discriminate patients with LV adverse remodeling (AUC = 0.65, *p* = 0.24, Supplementary Figure 1C). We identified that the relative expression of miR-874-3p in patients with and without adverse LV remodeling showed no statistical significance. These results indicated that we could discriminate patients with STEMI with and without adverse LV remodeling by measuring the levels of exosome miR-181a-3p.

## Discussion

In this study, we focused on the illustration of specific human plasma exosomal miRNAs in patients with STEMI postinfarction and their correlation to adverse left ventricular remodeling.

Our major finding was that exosomal miRNAs in the late stages of postinfarction were associated with myocardial injury and left ventricular expansion. However, they were less specific than traditional biomarkers such as NT-proBNP. In addition, the results ascertained that STEMI postinfarction induced profound changes in exosome miRNAs and, in part, metabolic perturbations such as the Ras signaling pathway, Rap1 signaling pathway, and phosphatidylinositol signaling system were key regulators in the progression of STEMI.

The progression of STEMI comprises a cascade of structural changes such as ventricular size and shape. Left ventricular remodeling results from non-infarcted myocardium in addition to augmented fibrosis and angiogenesis. In fact, they are controlled by a complexity of circulating miRNAs in a posttranscriptional manner. In the early stage of STEMI, most of the circulating miRNAs have been found and analyzed as potential biomarkers by *in vivo* and *in vitro* studies. However, there is no direct evidence to support that they were performed in patients prior to traditional indicators such as creatine kinase-MB (CK-MB) and NT-pro-BNP. Circulating miRNAs are considered to be novel therapeutic targets and an alternative approach in STEMI (24).

Compared to non-exosomal miRNAs, exosomal miRNAs are more sensitive, utilizing exosome isolation and miRNA amplification methods (25). In addition, exosome-miRNAs enriched in post-STEMI recovery may represent therapeutic targets for cardiomyocyte proliferation, cardiac vascularization, and antiapoptosis (24). It was reported that myocardium hypertrophy mediated by cardiac fibroblasts was associated with non-coding RNAs such as exosome-miR-21-3p (26). Massive parallel RNA sequencing results enable screening and analysis of entire miRNAs for a novel form of molecular medicine for heart disease (27).

Our data also revealed that exosome miRNA-21-3p was increased by 1.39-fold, but without remarkable significance (*p* = 0.059) in comparison with healthy subjects (data in Supplementary Files). Delivery of miRNA 21 to ischemic zones could rescue ischemic myocardium from the remodeling process (28). This result was consistent with a previous report, where upregulation of miR-21 protected cardiomyocytes from ischemic injury by inhibition of cell apoptosis (29).

In the myocardium remodeling phase of STEMI, non-infarcted myocardium gradually undergoes hypertrophy and expansion, leading to a reduction in left ventricular ejection fraction and poor prognosis. Clinical cardiovascular imaging such as MRI is also essential to assess the pathological changes of cardiomyocyte hypertrophy and contractility (30). However, changes of the circulating microenvironment in postinfarction STEMI could not be predicted. Our results predict that plasma exosome miRNA profile in patients with STEMI postinfarction was enriched in coagulation factors and fibroblast growth factors, which may promote microangiogenesis and prevent myocardial necrosis from forming ventricular aneurysms. It was reported that exosomes derived from a variety of cells such as myocardium, endocardium, and epicardium were widely involved in the development of cardiac repair and progression of inflammation (31).

It was reported that p53 expression was increased in patients with coronary artery disease (CAD), which was involved in the regulation of antiangiogenesis, cell death, regulation of metabolism, and cell cycle arrest (32). Downregulation of the p53 signaling pathway plays a critical role in the morphological changes of cardiomyocyte and myocardium remodeling, which includes cardiac hypertrophy and decreased heart function (33). Our results demonstrated that exosomal miRNAs targeting the p53 signaling pathway, which was consistent with previous reports; however, these results were only based on the GO ontology and the KEGG enrichment.

Expression levels of plasma miR-874-3p were downregulated in acute myocardial infarction (AMI) compared with healthy volunteers, which was consistent with this study (34). We also identified that there was a close relationship between miR-874-3p and glycolysis enzyme (LDH) in these patients. These findings indicated that miR-874-3p maintains a relative fall in either plasma or plasma exosome in different grades and stages of myocardial infarction. MiR-874-3p was recently found to target vascular endothelial growth factor A (VEGFA) (35), which binds to proangiogenic cells, including endothelial cells, hematopoietic stem cells, and monocytes (36). It was reported that cardiomyocyte necrosis results in cardiac myosin hypertrophy and leads to lower expression of exosome miR-874-3p. Overexpression of myosin in the heart could inhibit exosomal-miR-874 generation along with dysregulation of proangiogenic cells.

Indeed, levels of miR-181a-3p as well as miR-874-3p were reduced in atherosclerotic plaque (37). Increased miR-181a-3p expression may reduce the occurrence of metabolic syndrome and coronary artery diseases (CADs) (38). Additionally, microRNA-181a-3p is an essential regulator in vascular inflammation by targeting the nuclear factor-kappa B (NF-kB) pathway (39). Taken together, previous studies suggested that miR-181a-3p exerts an anti-inflammatory effect in CAD. Consistently, we identified that exosomal miR-181a-3p was downregulated in patients with LV adverse remodeling. These results indicated that exosome miRNAs may be linked to the progression of left ventricular myocardial hypertrophy and at least reflect the expansion of left ventricular hypertrophy.

An interpretation of exosome miRNAs profiles obtained from patients with STEMI 3–6 months postinfarction may reflect two destinies, one is recovery from vascular regeneration and another being irreversible heart failure. Exosome miRNAs released by non-infarcted myocardium could repair cardiac function in a paracrine manner following myocardial infarction (10). However, as this study had some limitations, we could not find out which exosome miRNAs were directly secreted from myocardial tissue postinfarction. We showed that exosome hsa-miR-181a-3p and exosome hsa-miR-874-3p expression levels postinfarction were lower than healthy volunteers. Bioinformatics analysis predicted that the top 20 downregulated miRNAs may play a critical role in fatty acid biosynthesis and metabolism. Dysfunction of lipid metabolism can result in premature atherosclerosis with its associated pathological change in coronary artery disease (CAD) (40). Recently, Agbu et al. uncovered that miR-33 can mediate glucose and lipid metabolism (41). Thus, enrichment of lipid metabolism may supplement the explanation of the role of exosomal miR-101-3p and miR-342-5p (Figure 3D) in CAD. Of note, most of the highest exosome miRNAs in high-throughput sequencing analysis contradicted the results in the validation group using qPCR. Our results suggested that most of them may play a negative role in left ventricular hypertrophy, which could be tested in transgenic mice and STEMI models using knockdown and overexpression methods. Currently, assessment of exosome miRNAs to predict LV remodeling remains premature. In addition, the sample size of 30 patients with STEMI and 30 healthy individuals was small; therefore, a large-scale study should be performed to validate these exosome miRNA candidates to predict the occurrence of STEMI and any existing association between exosome miRNAs and the early proinflammatory status during myocardial infarction (42).

## Conclusion

Circulating exosome miR-874-3p and miR-181a-3p were downregulated in patients with STEMI postinfarction. Exosome hsa-miR-181a-3p might play a potential role in development of LVR in patients with post-STEMI.

## Data Availability Statement

The data presented in this study are deposited in the GEO repository, accession number GSE 185729.

## Ethics Statement

The studies involving human participants were reviewed and approved by Sun Yat-sen Memorial Hospital, Sun Yat-sen University. The patients/participants provided their written informed consent to participate in this study.

## Author Contributions

YY designed the study and analyzed data. RG and KZ conducted experiments and acquired the data. RG and KZ wrote the manuscript and all the authors provided critical edits. All authors contributed to the article and approved the submitted version.

## Conflict of Interest

The authors declare that the research was conducted in the absence of any commercial or financial relationships that could be construed as a potential conflict of interest.

## Publisher’s Note

All claims expressed in this article are solely those of the authors and do not necessarily represent those of their affiliated organizations, or those of the publisher, the editors and the reviewers. Any product that may be evaluated in this article, or claim that may be made by its manufacturer, is not guaranteed or endorsed by the publisher.
