# Supplementary material for: Plasma Exosome miRNAs Profile in Patients With ST-Segment Elevation Myocardial Infarction
Source: Front Cardiovasc Med. 2022 Jun 15;9:848812. doi: 10.3389/fcvm.2022.848812 (PMC9240753; doi:10.3389/fcvm.2022.848812)
Supplement: Supplementary file 1 [file Data_Sheet_1.zip › Table 2.DOCX]

Table 2. List of candidate exosomal miRNAs (p < 0.05) from high through put sequence

| miRNA | Up/down | Fold change (IHD n=10 /Health n =6) |
| --- | --- | --- |
| hsa-miR-140-3p | down | *0.64* |
| hsa-miR-532-5p | down | *0.61* |
| hsa-miR-31-5p | down | *0.52* |
| hsa-miR-204-5p | down | *0.47* |
| hsa-miR-181a-3p | down | *0.64* |
| hsa-miR-874-3p | down | *0.50* |
| hsa-miR-132-5p | down | *0.47* |
| hsa-miR-20b-5p | down | *0.56* |
| hsa-miR-29a-5p | down | *0.42* |
| hsa-mir-106a-p3 | down | *0.44* |
| hsa-miR-106b-5p | down | *0.75* |
| hsa-miR-101-3p | down | *0.53* |
| hsa-miR-193a-3p | down | 0.49 |
| hsa-miR-4443 | down | *0.14* |
| hsa-miR-362-5p | down | *0.71* |
| hsa-miR-320 | down | *0.55* |
| hsa-miR-363-3p | down | *0.57* |
| PC-3p-56879_271 | down | *0.26* |
| hsa-miR-542-3p | down | *0.57* |
| hsa-miR-107 | down | *0.72* |
| hsa-miR-6815-5p | down | *0.36* |
| hsa-miR-873-5p | down | *0.28* |
| hsa-miR-542-3p | down | *0.57* |
| hsa-miR-107 | down | *0.72* |
| hsa-miR-6815-5p | down | *0.36* |
| hsa-miR-873-5p | down | *0.28* |
| hsa-miR-23a-3p | up | *1.33* |
| hsa-miR-224-5p | up | *3.15* |
| hsa-let-7d-3p | up | *1.34* |
| hsa-miR-940 | up | *4.48* |
| hsa-miR-3120-5p | up | *4.03* |
| hsa-miR-664a-3p | up | *1.21* |
| hsa-miR-34a-5p | up | *2.18* |
| hsa-miR-652-5p | up | *1.71* |
| hsa-miR-625-3p | up | *2.03* |
| hsa-miR-145-5p | up | *1.67* |
| hsa-miR-505-3p | up | *1.36* |
| hsa-miR-128-3p | up | *1.36* |
| hsa-miR-505-5p | up | *1.38* |
