# Supplementary material for: Plasma Exosome miRNAs Profile in Patients With ST-Segment Elevation Myocardial Infarction
Source: Front Cardiovasc Med. 2022 Jun 15;9:848812. doi: 10.3389/fcvm.2022.848812 (PMC9240753; doi:10.3389/fcvm.2022.848812)
Supplement: Supplementary file 1 [file Data_Sheet_1.zip › Table 1.DOCX]

Table 1. Characteristics of patients after STEMI 3-6 months (STEMI) and healthy volunteers (Health)

| Study varibales | Health (n = 30) | STEMI (n = 30) | p |
| --- | --- | --- | --- |
| Mean age, years(SD)  Males, N(%) | 32.37, (0.86)  25, 83 (%) | 64.71, (10.40)**  27, 90 (%) | 0.01  0.45 |
| Weight (kg) | 70.78, (1.70) | 65.64, (1.81) | 0.24 |
| Height (cm) | 165, (4.06) | 166, (1.13) | 0.83 |
| ALT | 18.67, (4.31) | 30.00, (7.03) | 0.26 |
| AST | 22.33, (2.90) | 38.67, (12.75) | 0.20 |
| Cr | 94, (5.53) | 131.5, (19.43) | 0.06 |
| Triglceride | 1.59, (0.35) | 1.43, (0.14) | 0.67 |
| Total cholesterol | 4.41, (0.21) | 4.50, (0.36) | 0.83 |
| HDL-C | 1.18, (0.04) | 0.93, (0.04) ** | 0.00 |
| LDL-C | 2.83, (0.14) | 3.01, (0.29) | 0.58 |
| NT-proBNP | <10 | 5985 | - |
| LDH | 160.9, (4.15) | 250.1, (18.67)** | 0.00 |
| LVEF (%) | 66.93, (0.67) | 41.5, (15.36) ** | 0.00 |
| LVEDV (ml) | 111.6, (2.49) | 149.4, (11.24) ** | 0.00 |

Abbreviation: ALT, Alanine aminotransferase; AST, Aspartate aminotransferase; Cr, Creatinine; HDL-C, High-density lipoprotein cholesterol; LDL-C, Low-Density lipoprotein cholesterol; LVEF, Left ventricular ejection fraction; LVEDV, LV end-diastolic volume. Comparison between STEMI patients and healthy volunteers. *p < 0.05, ** p < 0.01 versus healthy volunteers. (Data are presented as mean, (Std. Deviation)).
