# Supplementary material for: Plasma Exosome miRNAs Profile in Patients With ST-Segment Elevation Myocardial Infarction
Source: Front Cardiovasc Med. 2022 Jun 15;9:848812. doi: 10.3389/fcvm.2022.848812 (PMC9240753; doi:10.3389/fcvm.2022.848812)
Supplement: Supplementary file 1 [file Data_Sheet_1.zip › Table 1S .docx]

1. Xiang D, Xiang X, Zhang W, Yi S, Zhang J, Gu X, et al. Management and Outcomes of Patients With STEMI During the COVID-19 Pandemic in China. Journal of the American College of Cardiology. 2020;76(11):1318-24.

2. Zeymer U, Ludman P, Danchin N, Kala P, Laroche C, Sadeghi M, et al. Reperfusion therapies and in-hospital outcomes for ST-elevation myocardial infarction in Europe: the ACVC-EAPCI EORP STEMI Registry of the European Society of Cardiology. European heart journal. 2021.

3. Hausenloy DJ, Chilian W, Crea F, Davidson SM, Ferdinandy P, Garcia-Dorado D, et al. The coronary circulation in acute myocardial ischaemia/reperfusion injury: a target for cardioprotection. Cardiovascular research. 2019;115(7):1143-55.

4. Hanna A, Shinde AV, Li R, Alex L, Humeres C, Balasubramanian P, et al. Collagen denaturation in the infarcted myocardium involves temporally distinct effects of MT1-MMP-dependent proteolysis and mechanical tension. Matrix biology : journal of the International Society for Matrix Biology. 2021;99:18-42.

5. Rodriguez-Palomares JF, Gavara J, Ferreira-González I, Valente F, Rios C, Rodríguez-García J, et al. Prognostic Value of Initial Left Ventricular Remodeling in Patients With Reperfused STEMI. JACC Cardiovascular imaging. 2019;12(12):2445-56.

6. van der Bijl P, Abou R, Goedemans L, Gersh BJ, Holmes DR, Jr., Ajmone Marsan N, et al. Left Ventricular Post-Infarct Remodeling: Implications for Systolic Function Improvement and Outcomes in the Modern Era. JACC Heart failure. 2020;8(2):131-40.

7. Ibanez B, Rossello X. Left Ventricular Remodeling Is No Longer a Relevant Outcome After Myocardial Infarction. JACC Cardiovascular imaging. 2019;12(12):2457-9.

8. Dorn GW, 2nd. Novel pharmacotherapies to abrogate postinfarction ventricular remodeling. Nature reviews Cardiology. 2009;6(4):283-91.

9. Samouillan V, Martinez de Lejarza Samper IM, Amaro AB, Vilades D, Dandurand J, Casas J, et al. Biophysical and Lipidomic Biomarkers of Cardiac Remodeling Post-Myocardial Infarction in Humans. Biomolecules. 2020;10(11).

10. Sahoo S, Losordo DW. Exosomes and cardiac repair after myocardial infarction. Circulation research. 2014;114(2):333-44.

11. Pfeffer MA, Braunwald E. Ventricular remodeling after myocardial infarction. Experimental observations and clinical implications. Circulation. 1990;81(4):1161-72.

12. Mouton AJ, Rivera OJ, Lindsey ML. Myocardial infarction remodeling that progresses to heart failure: a signaling misunderstanding. American journal of physiology Heart and circulatory physiology. 2018;315(1):H71-h9.

13. Lu TX, Rothenberg ME. MicroRNA. The Journal of allergy and clinical immunology. 2018;141(4):1202-7.

14. Zheng D, Huo M, Li B, Wang W, Piao H, Wang Y, et al. The Role of Exosomes and Exosomal MicroRNA in Cardiovascular Disease. Frontiers in cell and developmental biology. 2020;8:616161.

15. Wang W, Zheng H. Myocardial Infarction: The Protective Role of MiRNAs in Myocardium Pathology. Frontiers in cardiovascular medicine. 2021;8:631817.

16. D'Alessandra Y, Devanna P, Limana F, Straino S, Di Carlo A, Brambilla PG, et al. Circulating microRNAs are new and sensitive biomarkers of myocardial infarction. European heart journal. 2010;31(22):2765-73.

17. Chimed S, van der Bijl P, Lustosa R, Fortuni F, Montero-Cabezas JM, Ajmone Marsan N, et al. Functional classification of left ventricular remodelling: prognostic relevance in myocardial infarction. ESC heart failure. 2022;9(2):912-24.

18. Li J, Zhang L, Wang Y, Zuo H, Huang R, Yang X, et al. Agreement in Left Ventricular Function Measured by Echocardiography and Cardiac Magnetic Resonance in Patients With Chronic Coronary Total Occlusion. Frontiers in cardiovascular medicine. 2021;8:675087.

19. Lang RM, Badano LP, Mor-Avi V, Afilalo J, Armstrong A, Ernande L, et al. Recommendations for cardiac chamber quantification by echocardiography in adults: an update from the American Society of Echocardiography and the European Association of Cardiovascular Imaging. Journal of the American Society of Echocardiography : official publication of the American Society of Echocardiography. 2015;28(1):1-39.e14.

20. Wen Z, Zhan J, Li H, Xu G, Ma S, Zhang J, et al. Dual-ligand supramolecular nanofibers inspired by the renin-angiotensin system for the targeting and synergistic therapy of myocardial infarction. Theranostics. 2021;11(8):3725-41.

21. Vlachos IS, Zagganas K, Paraskevopoulou MD, Georgakilas G, Karagkouni D, Vergoulis T, et al. DIANA-miRPath v3.0: deciphering microRNA function with experimental support. Nucleic acids research. 2015;43(W1):W460-6.

22. Morishima M, Kiriyama T, Miyagi Y, Otsuka T, Fukushima Y, Kumita SI, et al. Serial change in perfusion-metabolism mismatch after coronary artery bypass grafting. Annals of nuclear medicine. 2022;36(3):244-54.

23. Węgiel M, Wojtasik-Bakalarz J, Malinowski K, Surmiak M, Dziewierz A, Sorysz D, et al. Mid-regional pro-adrenomedullin and lactate dehydrogenase as predictors of left ventricular remodeling in patients with myocardial infarction treated with percutaneous coronary intervention. Polish archives of internal medicine. 2022;132(2).

24. Davidson SM, Andreadou I, Barile L, Birnbaum Y, Cabrera-Fuentes HA, Cohen MV, et al. Circulating blood cells and extracellular vesicles in acute cardioprotection. Cardiovascular research. 2019;115(7):1156-66.

25. Gallo A, Tandon M, Alevizos I, Illei GG. The majority of microRNAs detectable in serum and saliva is concentrated in exosomes. PloS one. 2012;7(3):e30679.

26. Bang C, Batkai S, Dangwal S, Gupta SK, Foinquinos A, Holzmann A, et al. Cardiac fibroblast-derived microRNA passenger strand-enriched exosomes mediate cardiomyocyte hypertrophy. The Journal of clinical investigation. 2014;124(5):2136-46.

27. Kaur K, Zangi L. Modified mRNA as a Therapeutic Tool for the Heart. Cardiovascular drugs and therapy. 2020;34(6):871-80.

28. Bejerano T, Etzion S, Elyagon S, Etzion Y, Cohen S. Nanoparticle Delivery of miRNA-21 Mimic to Cardiac Macrophages Improves Myocardial Remodeling after Myocardial Infarction. Nano letters. 2018;18(9):5885-91.

29. Gu H, Liu Z, Li Y, Xie Y, Yao J, Zhu Y, et al. Serum-Derived Extracellular Vesicles Protect Against Acute Myocardial Infarction by Regulating miR-21/PDCD4 Signaling Pathway. Frontiers in physiology. 2018;9:348.

30. Curley D, Lavin Plaza B, Shah AM, Botnar RM. Molecular imaging of cardiac remodelling after myocardial infarction. Basic research in cardiology. 2018;113(2):10.

31. Chistiakov DA, Orekhov AN, Bobryshev YV. Cardiac Extracellular Vesicles in Normal and Infarcted Heart. International journal of molecular sciences. 2016;17(1).

32. Men H, Cai H, Cheng Q, Zhou W, Wang X, Huang S, et al. The regulatory roles of p53 in cardiovascular health and disease. Cellular and molecular life sciences : CMLS. 2021;78(5):2001-18.

33. Nomura S, Satoh M, Fujita T, Higo T, Sumida T, Ko T, et al. Cardiomyocyte gene programs encoding morphological and functional signatures in cardiac hypertrophy and failure. Nature communications. 2018;9(1):4435.

34. Yan Y, Song X, Li Z, Zhang J, Ren J, Wu J, et al. Elevated levels of granzyme B correlated with miR-874-3p downregulation in patients with acute myocardial infarction. Biomarkers in medicine. 2017;11(9):761-7.

35. Huang Y, Han Y, Guo R, Liu H, Li X, Jia L, et al. Long non-coding RNA FER1L4 promotes osteogenic differentiation of human periodontal ligament stromal cells via miR-874-3p and vascular endothelial growth factor A. Stem cell research & therapy. 2020;11(1):5.

36. Staudacher DL, Preis M, Lewis BS, Grossman PM, Flugelman MY. Cellular and molecular therapeutic modalities for arterial obstructive syndromes. Pharmacology & therapeutics. 2006;109(1-2):263-73.

37. Su Y, Yuan J, Zhang F, Lei Q, Zhang T, Li K, et al. MicroRNA-181a-5p and microRNA-181a-3p cooperatively restrict vascular inflammation and atherosclerosis. Cell death & disease. 2019;10(5):365.

38. Hulsmans M, Sinnaeve P, Van der Schueren B, Mathieu C, Janssens S, Holvoet P. Decreased miR-181a expression in monocytes of obese patients is associated with the occurrence of metabolic syndrome and coronary artery disease. The Journal of clinical endocrinology and metabolism. 2012;97(7):E1213-8.

39. Sun X, Icli B, Wara AK, Belkin N, He S, Kobzik L, et al. MicroRNA-181b regulates NF-κB-mediated vascular inflammation. The Journal of clinical investigation. 2012;122(6):1973-90.

40. Knebel B, Müller-Wieland D, Kotzka J. Lipodystrophies-Disorders of the Fatty Tissue. International journal of molecular sciences. 2020;21(22).

41. Agbu P, Carthew RW. MicroRNA-mediated regulation of glucose and lipid metabolism. Nature reviews Molecular cell biology. 2021;22(6):425-38.

42. Dickhout A, Koenen RR. Extracellular Vesicles as Biomarkers in Cardiovascular Disease; Chances and Risks. Frontiers in cardiovascular medicine. 2018;5:113.
